# Dynamic Changes in Volatile Flavor Compounds, Amino Acids, Organic Acids, and Soluble Sugars in Lemon Juice Vesicles during Freeze-Drying and Hot-Air Drying

**DOI:** 10.3390/foods11182862

**Published:** 2022-09-15

**Authors:** Huanxiong Xie, Ru Zhao, Chunju Liu, Yulong Wu, Xiaojie Duan, Jiaqi Hu, Feifei Yang, Haiou Wang

**Affiliations:** 1 Key Laboratory of Modern Agricultural Equipment, Nanjing Research Institute for Agricultural Mechanization, Ministry of Agriculture and Rural Affairs, Nanjing 210014, China; 2 School of Food Science, Nanjing Xiaozhuang University, Nanjing 211171, China; 3 Institute of Agro-Product Processing, Jiangsu Academy of Agricultural Sciences, Nanjing 210014, China; 4 College of Food Science, Shenyang Agricultural University, Shenyang 100866, China

**Keywords:** lemon juice vesicles, vacuum freeze-drying, hot-air drying, volatile flavor compounds, nonvolatile flavor compounds

## Abstract

Lemon juice vesicles have abundant flavor components that can undergo complex changes during drying. Three drying methods, including integrated freeze-drying (IFD), conventional freeze-drying (CFD), and hot-air drying (AD), were studied to determine their effects on the dynamic changes in the flavor compounds in lemon juice vesicles. Compared with the fresh samples, the final dried samples that underwent IFD, CFD, and AD lost seven, seven, and six volatile flavor compounds and three, four, and five amino acids, respectively; the order of the loss ratios with respect to the volatile compound content was: 82.73% in CFD > 71.22% in IFD > 28.78% in AD. AD resulted in the highest total amino acid content (10.83 ± 0.20 mg/g), which was 1.39 and 5.54 mg/g higher than that of IFD and CFD, respectively; CFD resulted in the highest total organic acid content (45.94 ± 0.34 mg/g), which was 8.01 and 7.87 mg/g higher than that of IFD and AD, respectively; and AD contributed to the highest total soluble sugars (17.12 ± 0.20 mg/g), which was 1.24 and 1.49 mg/g higher than that of IFD and CFD, respectively. A correlation analysis demonstrated that most of the amino acids and the soluble sugars were closely related to the profiles of the volatile compounds in the lemon juice vesicles during drying.

## 1. Introduction

Lemons (*Citrus limon*) are cultivated in many countries that have temperate summers and mild winters. They have become one of the most popular fruits worldwide due to their unique flavor and abundant nutrients (such as essential oils, vitamins, amino acids, and phenolics) [1,2,3]. Generally, fresh fruits comprise most of the commercial sales of lemons but, because they are hard to preserve, selling the fresh fruits can easily cause huge losses. Furthermore, the fresh pulps of lemons (mainly composed of juice vesicle tissue) are hardly eaten directly due to their unacceptably sour taste. Instead, fresh lemons are usually processed into dried slices or powders, which are brewed in hot water or used as food additives. In recent years, dried lemon slices have become a popular healthy drink for many consumers of different ages [4].

Recently, different drying techniques have been used to dry lemons to investigate their drying process parameters, drying performance, and the quality of the final product. Wang et al. employed pulsed-vacuum-drying technology to dry lemon slices, and the influences of the drying temperature on the drying kinetics, shrinkage, rehydration ratio, microstructure, and color parameters were investigated [4]. Torki-Harchegani et al. revealed the effects of drying temperature on the drying behavior and mass transfer parameters of lemon slices dried by a laboratory air-ventilated oven dryer [5]. García-Pérez et al. found that the application of power ultrasound during the convective drying of lemon peels improved the effective moisture diffusivity, and the improvement was linearly proportional to the acoustic power density applied [6]. Chen et al. confirmed that drying lemon slices using a closed-type solar dryer presented a better performance than using the hot air-dried ones [7]. Xu et al. compared the effects of four drying methods, namely, hot-air drying (AD), microwave drying, vacuum drying, and microwave vacuum drying, on the quality of lemon slices, and found that microwave vacuum drying had a better effect on the sensory perception and nutritional properties of the lemon slices [8]. Salehi et al. investigated the modeling of moisture loss kinetics and color changes on the surface of lemon slices during combined infrared-vacuum drying [9]. Zhang et al. found that hot-air and infrared drying resulted in significant decreases in the yield and the antioxidant and antibacterial activities of essential oils in the lemon peel [10]. Hot-air drying (AD) and vacuum freeze-drying (FD) are the most common processing methods for drying lemon slices in the food industry.

Although AD is time-consuming and causes serious deterioration of the products’ quality, it is still widely used for food drying due to its low capital cost and its simple operation [11]. FD can retain the original nutritional content and the color, taste, and shape of the raw materials to the greatest extent due to the high-intensity vacuum and low temperature employed. Therefore, it has been considered the best method for producing high-quality dehydrated foods, while it is also the most expensive method due to its high energy consumption and the high costs of both its operation and maintenance [12,13]. The FD process of foods mainly involves the following stages: pre-treating, freezing, freeze-drying (primary drying and secondary drying), and packaging. Freezing is viewed as an indispensable and crucial stage of the FD process. In conventional freeze-drying (CFD), the freezing process is traditionally conducted via contact plate freezing or air blast freezing under atmospheric pressure. Additionally, vacuum freezing (VF) under vacuum conditions is used as the freezing method in integrated freeze-drying (IFD), which can simplify the assembly line and the operation process of CFD in large-scale production and reduce its production cost [14].

Lemons have been recognized as a type of fruit with abundant flavor components, which are composed of complex combinations of volatile flavor compounds (mainly alkenes, alcohols, aldehydes, esters, and ketones) and nonvolatile flavor compounds (mainly amino acids, organic acids, and soluble sugars) [15,16]. Lemons are mainly composed of flavedo, albedo, juice vesicles, segment walls, and seeds, presenting a heterogeneous histological structure and different flavor compounds [17,18]. Juice vesicles, as the major edible tissue of the lemon (accounting for more than 60% of the fruit in both volume and weight), are the main commercial products of dried lemon slices or powders. However, to the best of our knowledge, no studies have been carried out on the dynamic changes in the flavor compounds during the drying process of lemon juice vesicles.

Therefore, the aim of this study is to evaluate the changes in the main volatile compounds and nonvolatile compounds (amino acids, organic acids, and soluble sugars) of lemon juice vesicles during the drying processes of IFD, CFD, and AD. The current study could provide more data and information for selecting the most suitable drying technology and for controlling the flavor quality of lemon juice vesicles during drying processes.

## 2. Materials and Methods

### 2.1. Materials and Regents

Fresh lemons (Eureka, Anyue, Sichuan, China) were purchased from a local supermarket in Nanjing. A standard sample of Cyclohexanone (>99.9%; C6–C25) was purchased from Sigma-Aldrich Trading Co., Ltd. (Shanghai, China). Sodium chloride, amino acid standard, o-phthalaldehyde (OPA), acetonitrile, methanol, 3-mercaptopropionic acid, boric acid, potassium dihydrogen phosphate, petroleum ether (30–60 °C), absolute ethanol, phosphoric acid, hydrochloric acid, ammonia water, ether, pyrogallic acid, ethanol (95%), and other reagents were purchased from Sinopharm Chemical Reagent Co., Ltd. (Beijing, China).

### 2.2. Processes Involved in Different Drying Methods

Lemons of uniform size (major axis diameter of 70 ± 5 mm and minor axis diameter of 60 ± 5 mm) were randomly selected as the experimental materials. These lemons had a bright color and were damage-free. The fresh lemons were cleaned using tap water and cut into round slices (1 cm thick), according to the direction perpendicular to the fruit’s axis. The flavedo, albedo, segment walls, and seeds were then removed, and the juice vesicles were made into unit samples with a mass of 2.00 ± 0.01 g. If the unit samples were too large and non-uniform, the drying time would be long and inconsistent. Thus, a relatively standard sample with a mass of about 2 g was more conducive to maintaining consistent drying rates for the unit samples in the same drying method. Thereafter, IFD, CFD, and AD treatments were carried out. In order to ensure the wet-base moisture content of the unit samples below 5%, the drying times of IFD, CFD, and AD were set as 12 h, 12 h, and 6 h, respectively. During the drying process, the test samples were taken out for the determination of relevant volatile and nonvolatile compounds. The specific drying and sampling methods are as follows:

IFD: The vacuum freeze dryer (SCIENTZ-50F, Ningbo Scientz Biotechnology Co., Ltd., Ningbo, China) was turned on in advance to reduce the temperature of the cold trap to −40 °C. The unit was then placed above the juice vesicle samples in the freeze dryer, which underwent vacuuming and freezing for 0.5 h. During this time, the moisture of the lemon juice vesicles quickly evaporated and simultaneously removed a large quantity of heat from the samples. The samples were subsequently frozen and crystallized. Thereafter, the samples were heated according to the pre-set automatic controlling procedure with the temperature–duration times of −20 °C for 2 h, −10 °C for 2 h, 0 °C for 2 h, 10 °C for 2 h, 20 °C for 1 h, 30 °C for 1 h, 40 °C for 1 h, and 50 °C for 1 h; meanwhile, the vacuum pressure during FD was maintained below 50 pa. The schedule of the sampling time during the drying process comprised vacuum freezing for 0.5 h (IF for 0.5 h), freeze-drying for 4 h (IFD for 4 h), freeze-drying for 8 h (IFD for 8 h), and freeze-drying for 12 h (IFD for 12 h).

CFD: The above-mentioned juice vesicle samples were frozen at −40 °C under normal pressure for 4 h before being freeze-dried in the vacuum freeze dryer. The heating program of FD was the same as that of IFD. During the drying process, the schedule of the sampling time comprised freezing for 4 h at atmospheric pressure (CF for 4 h), freeze-drying for 4 h (CFD for 4 h), freeze-drying for 8 h (CFD for 8 h), and freeze-drying for 12 h (CFD for 12 h).

AD: The juice vesicle samples were placed into a hot air-drying oven (DHG-9070, Shanghai Xinmiao Medical Equipment Co. Ltd., Shanghai, China) to dry at 60 °C. The schedule of the sampling time during the drying process comprised air drying for 2 h (AD for 2 h), air drying for 4 h (AD for 4 h), and air drying for 6 h (AD for 6 h).

### 2.3. Determination of Related Indicators

#### 2.3.1. Determination of Volatile Compounds

One unit sample with 2.00 ± 0.01 g of juice vesicles was taken as the testing sample for the measurement of volatile compounds and the subsequent indicators. The volatile compounds in the lemon juice vesicle samples were extracted and separated using headspace solid-phase microextraction technology. The specific process involved the whole unit sample being cut into small uniform pieces; thereafter, distilled water was added to increase the volume to 20 mL, and the mixture was homogenized for 2 min at a speed of 9000 r/min using a homogenizer (FJ200-SH, Shanghai Huxi Industrial Co., Ltd., Shanghai, China). A 5 mL portion of the homogenized sample solution was placed into a 15 mL headspace vial for extraction, and 2 μL of cyclohexanone was added to the sample solution as an internal standard in the quantitative analysis of GC-MS. The CAR/PDMS/DVB solid-phase microextraction needle (50/30 um divinylbenzene carboxene-poly (dimethylsiloxane), Supelco, Bellefonte, PA, USA) was inserted into the headspace of the sample vial, and the sample vial was placed in a water bath at 60 °C for 30 min. Thereafter, the extraction needle was removed and inserted into the GC-MS injector, where it was desorbed at 250 °C for 5 min; simultaneously, the instrument began data collection.

The volatile compounds in the testing samples were determined using a GC-MS (GCMS-QP2010, Shimadzu Corporation, Japan) equipped with an HP-5 ms (30 m × 0.25 mm, 0.25 μm) chromatographic column. The specific heating program involved the initial oven temperature being at 50 °C, which was then held for 5 min, increased in 10 °C/min increments to 230 °C, and then held for another 5 min. Helium carrier gas was used at a flow rate of 1 mL/min. The temperature at the injection port was 250 °C, and the injection mode was manual. Mass spectrometric detection parameters were set as follows: ionization method—EI, electron energy—70 eV, ion source temperature—230 °C, MS quadrupole temperature—150 °C, mass scanning range mass-to-charge ratio (m/z)—30–450, and a scanning rate of 5.27 times/s. The determination results were qualitatively matched by a computer and the standard spectral library, and the identification results were based on the results with a similarity greater than 70, which were combined with the relevant reports and literature to determine the chemical components of the corresponding volatile flavor compounds [1,4,19,20,21,22].

Since the mass of the unit sample was continuously reduced during the drying process, the GC-MS results were presented as milligrams of volatile compound per gram of the original fresh unit sample (mg/g FW). This could facilitate the comparison of the volatile compound content in the lemon juice vesicles at different drying stages. In the test, the fresh lemon juice sample was used as the control. The measurement was repeated three times at each sampling stage during the undertaking of the three drying methods. The test results were calculated as the “mean ± standard deviation”.

#### 2.3.2. Microstructure Analysis via Scanning Electron Microscopy

The final dried product samples at IFD for 12 h, CFD for 12 h, and AD for 6 h underwent microstructure analysis using a scanning electron microscope (SEM, EVO-LS10, Cambridge, Germany). First, cross-sectional observed samples were obtained by naturally fracturing the dried samples with the aid of instant freezing with liquid nitrogen. Thereafter, the observed samples were coated with gold under vacuum conditions, and the outer surface was observed on the SEM using an accelerating voltage of 10 kV. Typical apparent photographs of the cross-sectional observed samples were taken for the microstructure analysis of the three dried samples.

#### 2.3.3. Determination of Free Amino Acids

Determination of the free amino acid content was conducted according to the method by Gao et al. with slight modifications [23]. The specific process was as follows: one unit sample of juice vesicles was cut into small pieces of uniform size and ground in an ice bath. An amount of 10 mL of deionized water was added to this sample, and the mixture was placed in a 50 mL centrifuge tube, homogenized at 15,000 r/min for 2 min, which then underwent ultrasonic treatment for 30 min. Finally, this mixture was centrifuged at 8000 r/min for 15 min, and the supernatant fraction was taken for testing.

The amino acid samples were precolumn-derivatized with OPA reagent solution. The derivatization reagent OPA was prepared by adding 1.5 mL of 0.4 mol/L boric acid buffer (pH 10.2) and 15 µL of 3-mercaptopropionic acid to 15 mg OPA, which was then stored at 4 °C. Subsequently, 50 μL of borate buffer was added 10 μL of the amino acid sample, which after 0.5 min was mixed with 10 μL of OPA and 640 μL of ultrapure water. This solution was then filtered with a 0.45 μm filter membrane, and the derivatized solution sample was prepared. The amino acid components and content in the derivatized solution sample were determined using a high performance liquid chromatograph (HPLC, HP1200, Agilent, Santa Clara, CA, USA) equipped with a ZORBAX Eclipse-AAA chromatographic column (4.6 × 150 mm, 3.5 μm, Agilent, USA). The mobile phase ratio was 45:45:10 of acetonitrile: methanol: water (*v*/*v*/*v*), the injection volume was 20 μL, and the column temperature was 40 °C.

To facilitate the comparison of the amino acid measurements during the drying processes, the concentration of each component was also presented as milligrams of amino acid per gram of the original fresh unit sample (mg/g FW). The measurement was repeated three times at each sampling stage during the three drying methods. The test results were calculated as the “mean ± standard deviation”.

#### 2.3.4. Determination of Organic Acids

According to the Chinese national standard GB5009.157-2016, the “national food safety standard for the determination of organic acids in food”, the specific method for determining the organic acids in a compound was applied as follows: One unit sample of juice vesicles was cut into small uniform pieces in a 50 mL centrifuge tube. To this, 20 mL of deionized water was added, and the mixture was homogenized at a speed of 15,000 r/min for 2 min and then centrifuged at 4000 r/min for 5 min. The supernatant fraction was placed into a 25 mL of volumetric flask, and the residue was extracted with 20 mL of deionized water once again. The second extraction’s supernatant was combined in the same 25 mL volumetric flask, and the volume was adjusted to the mark of 25 mL with deionized water.

The HPLC (HP1200, Agilent, USA) was used for the determination of organic acids using a VP-ODSC18 column (250 × 4.6 mm, 5 um, Agilent, USA), and the specific measurement conditions included a column temperature of 30 °C, a mobile phase of 0.02 mol/L of KH_2_PO_4_ buffer solution (pH 3.0), a flow rate of 1.0 mL/min, an injection volume of 20 μL, and a wavelength of 215 nm. The concentration of each component was also presented as milligrams of organic acid per gram of the original fresh unit sample (mg/g FW). The measurement was repeated three times at each sampling stage when the three drying methods were being carried out. The test results were calculated as the “mean ± standard deviation”.

#### 2.3.5. Determination of Soluble Sugars

The concentration of soluble sugars was measured according to the Chinese national standard GB5009.8-2016, the “Determination of fructose, glucose, sucrose, maltose and lactose in food”, and the method of Pei et al. with a slight modification [24]. The specific method was as follows: one unit sample of juice vesicles was cut into small pieces of uniform size in a 50 mL centrifuge tube. To this, 20 mL of water was added, and the mixture was homogenized at a speed of 15,000 r/min for 2 min, and then sonicated for 30 min at 20 °C in a sonication bath. Thereafter, the mixture was centrifuged at 10,000 r/min for 15 min. The residue was added to 4 mL of distilled water and again was extracted twice, and the three periods of extraction solutions were combined and filtered with a 0.45 μm filter membrane; thus, the solution sample used in the determination of the soluble sugars was prepared.

The determination of soluble sugar concentration was carried out using an HP1200 HPLC equipped with a Series200 amine-based column (250 × 4.6 mm, 5 µm). The column temperature was 40 °C, the mobile phase ratio was acetonitrile: water (70:30, *v*/*v*), the flow rate was 1.0 mL/min, the injection volume was 20 mL, the temperature of the refractive index detector was 40 °C, the temperature of the drift tube of the evaporative light scattering detector was 80–90 °C, the nitrogen pressure was 350 kPa, and the impactor was closed. The calculation method for the measurement result was identical to that described in Section 2.3.3.

### 2.4. Statistical Analysis

ANOVA and Tukey’s HSD test of the SPSS statistics software (version 19.0; IBM, Chicago, IL, USA) were used to analyze the data. Statistical significance of the data was defined when *p* < 0.05. Principal component analysis (PCA), hierarchical cluster analysis (HCA), and Pearson’s correlation analysis were used for comparing and discriminating the data from the multivariate dataset using OriginPro9 software (Origin Lab., Northampton, MA, USA).

## 3. Results and Discussion

### 3.1. Changes in Volatile Flavor Compounds during the Drying Processes

#### 3.1.1. Volatile Flavor Compounds and Content in Lemon Juice Vesicles during Drying Process

The loss of the volatile compounds in foods caused by drying has been widely reported. Chin et al. found that the FD process caused a reduction in the amount of major aroma volatiles in durian pulp, which ranged from 71.5% to 97.2% [25]. The total loss ratios of the volatile compounds of freeze-dried foods have been reported in some studies, including 50% in coffee extract [26], 37.5% in banana slices [27], and 55% in breadcrumbs [28]. Rajkumar et al. found increases and decreases of different extents in concentrations in key volatile compounds of air-dried and freeze-dried cabbages [29]. Venskutonis et al. reported that the retention of the volatile compounds in freeze-dried thyme and sage was even lower than that with hot-air drying under mild conditions [30]. The characteristics of volatile compounds in dried fruits and vegetables are influenced by chemical and physical changes. This has been extensively studied by the relevant scholars.

In our experiments, the components and content of the volatile flavor compounds in lemon juice vesicles during the processes of IFD, CFD, and AD are shown in Table 1. A total of 22 volatile flavor compounds were detected in the fresh samples and in the samples at different drying stages of the three methods. These were mainly composed of alkenes (five types), alcohols (seven types), aldehydes (six types), esters (three types), and ketones (one type). Particularly, diisobutyl phthalate was not detected in either the fresh samples or experimental samples during AD, but it was newly generated during both IFD and CFD. The top six volatile compounds with relatively higher contents in fresh samples, in decreasing order, were D-limonene, α-terpineol, citral, γ-terpinene, nerol, and neral.

The number and content of the volatile compounds in food are closely related to the food’s flavor quality. In terms of IFD, the types of volatile flavor compounds detected at the stages IF 0.5 h, IFD 4 h, IFD 8 h, and IFD for 12 h were 20, 18, 14, and 15, respectively. The types detected at the four stages (CF for 4 h, CFD for 4 h, CFD for 8 h, and CFD for 12 h) in CFD were 16, 14, 13, and 15, respectively, and those detected at stages AD for 2 h, AD for 4 h, and AD for 6 h were 18, 15, and 16, respectively. No obvious differences were observed in the numbers of volatile compounds of the dried lemon juice vesicles at the end of the drying process of the three methods. Compared with the fresh sample, lemon juice vesicles dried via IFD, CFD, and AD lost seven, seven, and six volatile flavor compounds, respectively, which weakened its original flavor quality.

The original volatile compounds in the fresh samples are partially retained in the dried product through many kinds of drying methods. Regarding the physical changes, two major theories have been widely accepted to explain the retention mechanism of volatile compounds in foods. The first one is the selective diffusion theory of Thijssen and Rulkens, which explains that the diffusion coefficients of other substances are much lower than the diffusion coefficient of water as dehydration progresses [27,31,32]. The second one is the microregion entrapment theory proposed by Flink and Karel, which includes the physical encapsulation of volatile compounds by macromolecules upon the formation of microregions during the drying process [33,34]. The retention of volatiles in dried samples is controlled by complicated factors including the sample dimensions, sample matrix, type of solid, initial content of solid, initial volatile concentration, and drying rate [35].

In light of the changes in the content of the data, it can be said that the content of the volatile flavor compounds gradually decreased as the processes of IFD and CFD proceeded. Generally, a similar descending trend was observed in the content of the top six volatile compounds, the subtotal contents of every compound category, as well as the total content of all the compounds during IFD and CFD. During the two FD processes, the loss of volatiles in the early stage of freeze-drying was much faster than that in the later stage of drying, which was consistent with the results from Krokida et al. [33] and Kompany et al. [36,37]. This could be explained by the selective diffusion theory and the microregion entrapment theory. During the freezing of juice vesicles, the crystallization of water results in the formation of scores of microregions entrapping highly concentrated solutions of volatile compounds and carbohydrates [27]. As dehydration progresses during FD, the local moisture content within the microregions decreases, the molecular association of carbohydrates occurs via hydrogen bonds, and the diffusing resistance of volatiles within the microregions increases; thus, volatile loss decreases. As the moisture content in the local microregions reaches a critical level, the microregions are sealed; thereafter, volatile loss ceases. In addition, at the initial temperatures of FD, these volatile compounds have higher vapor pressures than ice, resulting in the rapid evaporation of the volatile compounds from the surface and the interior of the frozen material [33]. Therefore, the volatile compounds in the juice vesicles were evaporated at a faster rate during the early stages of FD than the last period of drying.

**Figure 1:**
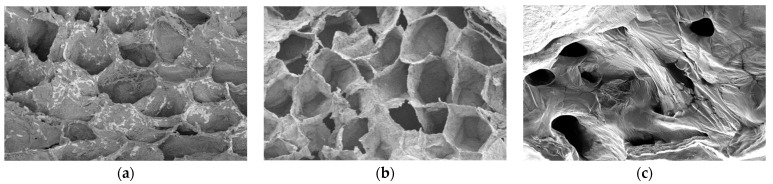
SEM images (at 300 magnification) of the final dried samples in the three drying methods. (**a**): IFD for 12 h; (**b**): CFD for 12 h; (**c**): AD for 6 h.

Concerning the three drying methods, juice vesicles dried via AD were detected with much higher amounts of volatile compounds than IFD and CFD. The loss ratios of the volatile compounds in the products with dried juice vesicles, relative to those in the fresh samples, were ranked in increasing order as 28.78% at AD for 6 h, 71.22% at IFD for 12 h, and 82.73% at CFD for 12 h, showing significant differences (*p* < 0.05). Unexpectedly, the two FD methods result in a much greater loss of volatile compounds than AD. At the beginning of AD, the juice vesicles underwent an intensive dehydration process due to the high temperature convection drying, and volatile compounds were quickly lost. As the AD progressed, sugars and other solutes diffused to the surface of samples. When the moisture content fell below a critical value, an impermeable, thin layer was formed on the surface and the inner microstructure of the juice vesicle samples in AD for 6 h (Figure 1c) severely collapsed and shank, subsequently appearing to be much denser and harder than those in IFD for 12 h (Figure 1a) and CFD for 12 h (Figure 1b). This can impede the diffusion of volatile compounds and cause the retention of more volatile compounds in the final products [27]. The dehydration of the FD was relatively mild and lasted for a long time, forming relatively loose and plump porous microstructures in the samples (Figure 1a,b), which was consequently a process that was beneficial to the diffusion of the volatile compounds. During FD, volatile flavor compounds could escape from cellular tissues due to the action of the prolonged high-vacuum conditions and the sample temperature that was near 50 °C around the drying end, leading to the significantly greater loss of volatile compounds with this process than with AD.

By comparing the synchronous stages of the two FD methods, it can be seen that IFD caused a significantly reduced loss in the total content of all the volatile compounds, indicating that the IFD method had obvious advantages with respect to the retention of volatile compounds during drying when compared with CFD, which was consistent with the results of Wang et al. and Xie et al. [38,39]. Based on the selective diffusion theory and the microregion entrapment theory, we deduced that the 0.5 h of vacuum freezing of IFD can rapidly produce smaller ice crystals in the sample tissues, and then sever the intercellular network channels for the diffusion of the volatile compounds. Moreover, the concentrations of macromolecular components (cellulose, hemicellulose, and pectin) in the cell walls increased rapidly due to the moisture loss that occurred during the 0.5 h of vacuum freezing. This enhanced the diffusing resistance of the volatile compounds, which were more conducive to the action of the microregion entrapment and the retention of volatile compounds in the sample tissues during the IFD process. By comparing the microstructures of the dried samples, as shown in Figure 1a,b, we can observe that a denser porous network structure was formed in the IFD-dried samples, and the microregions encapsulated by cell wall pores were relatively intact, which was more beneficial to the retention of flavor compounds than what had occurred in the CFD-dried samples. It has been proven by the studies on the FD of various materials—including spearmint [40], apricots [41], and basil [42]—that the retention of volatiles is closely related to the integrity of the microstructure in cellular tissues during drying, and that the freezing and drying conditions may change the microstructures of cells and affect the diffusion of volatiles in tissues. Regarding the chemical changes, the loss of volatile flavor compounds after dehydration may be due to the inactivation of volatile-forming enzymes and the loss of their precursors [25,32]. Nevertheless, the introduction of new compounds during dehydration may be attributed to the autoxidation of unsaturated fatty acids and thermal decomposition, and/or the initiation of the Maillard reaction, which are both rather complex [25,32]. Complicated changes were observed in the content of volatile compounds during AD. Compared with the fresh sample, some volatile compounds (including β-myrcene, terpinen-4-ol, (-)-terpinen-4-ol, nonanal, perilla aldehyde, and lavandulyl acetate) were significantly increased during or after 6 h of AD, whereas the top six higher volatile compounds were all significantly decreased after AD for 6 h. The total content of volatile compounds during the AD process presented a trend of first decreasing (at AD for 2 h) and then increasing (at AD for 4 h and AD for 6 h). The juice vesicle samples during the AD process were in direct contact with an environment with a high temperature and sufficient oxygen; thus, lipid autoxidation reactions and Maillard reactions within the samples were easily initiated by initiators such as heat, metallic ions, and light, and were influenced by complicated factors such as the temperature, reaction time, water activity, precursor structure, and enzyme activity [32]. The lipid autoxidation reactions and Maillard reactions during AD would lead to a considerable effect on the volatile flavor of the dried samples. However, within the scope of our present studies and knowledge, it was very difficult to explain the reasons for the chemical changes (the loss, increase, and new introduction) of the volatile compounds.

#### 3.1.2. PCA, HCA, and Pearson’s Correlation Analysis on the Volatile Flavor Compounds

**Figure 2:**
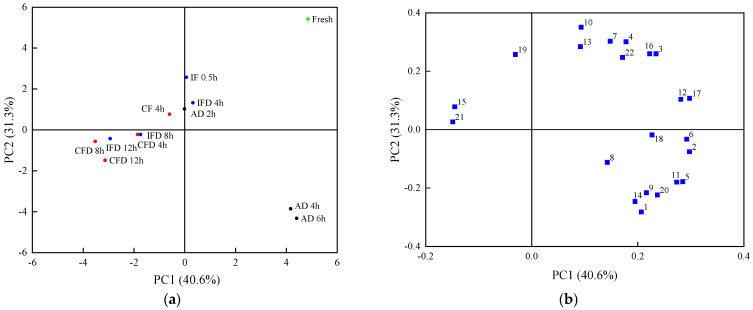

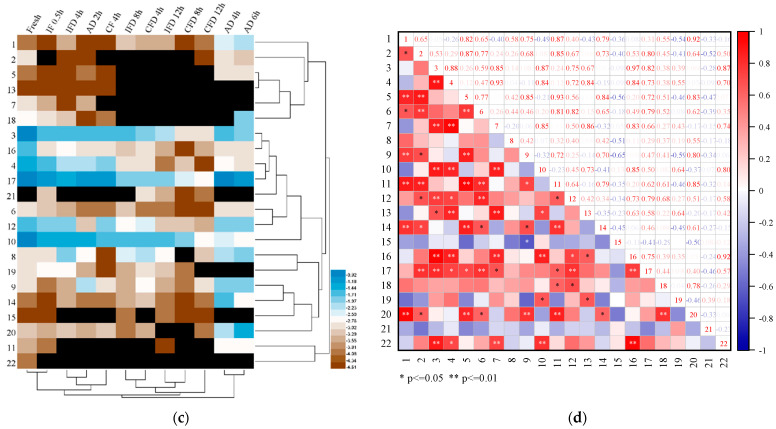
Principal component analysis, hierarchical cluster analysis, and Pearson’s correlation analysis based on the content of volatile flavor compounds in the samples during the drying processes. Numbers 1~22 are the corresponding codes for the 22 kinds of volatile compounds in Table 1. (**a**): Score plot of principal component analysis of the different drying stages; (**b**): Score plot of principal component analysis of the volatile flavor compounds; (**c**): Heatmap of hierarchical cluster analysis; (**d**): Heatmap of Pearson’s correlation analysis.

A PCA was used to analyze the effect of drying processes on the volatile compound profiles of the juice vesicles. Figure 2a showed that 71.9% of the total volatile compound variance was described by the first and second principal components (PC1, 41.6%; PC2, 32.1%). The PCA results in Figure 2a demonstrated that the samples at different drying stages could be well distinguished according to the scores of PC1 and PC2. Fresh samples could be well defined according to the maximum positive PC1 and PC2. The samples of the AD for 4 h and AD for 6 h could be well-separated by the maximum positive PC1 and negative PC2. The samples of IF for 0.5 h, IFD for 4 h, AD for 2 h, and CF for 4 h could be defined by a low positive PC2 score and a low PC1 score, and the samples of other stages could be separated by low negative PC2 and PC1 scores. In addition, 22 volatile compounds could be distinguished based on the scores of PC1 and PC2 (Figure 2b).

An HCA was conducted to specify the differences among the drying stages and volatile compounds. The samples and volatile compounds were classified based on their similarity (Figure 2c). The samples at different drying stages fell into three main clusters, which were almost consistent with the PCA results in Figure 2a. The fresh, IF for 0.5 h, IFD for 4 h, AD for 2 h, and CF for 4 h samples were in the first cluster; the samples of IFD for 8 h, CFD for 4 h, IFD for 12 h, CFD for 8 h, and CFD for 12 h were in the second cluster; and the samples of AD for 4 h and AD for 6 h were in the third cluster. The volatile compounds could be divided into four principal cluster groups. The first cluster included compounds 1, 2, 5, 13, 7, and 18. The second cluster included compounds 3, 16, 4, 17, 21, 6, 12, and 10. The third cluster comprised compounds 8, 19, 9, 14, 15, and 20, and the fourth cluster included compounds 11 and 22.

A correlation analysis was used to investigate the correlation coefficients of volatile compounds during the drying processes. Pearson’s correlation coefficients of the 22 volatile compounds are shown in Figure 2d. Among them, red and blue represent the positive and negative correlations in the pairs, respectively, and the darker colors denote stronger correlations. In terms of the pairs of relations, the pairs involved in compounds 2, 3, 4, 5, 11, 12, 16, 17, and 20 were observed with relatively more significant positive correlations (8–10 pairs; *p* < 0.05), demonstrating that the changes in these compounds were closely related to each other. However, there was no significant correlation with the pairs involved in compound 21. The pairs involved in compounds 8, 13, 15, 18, 19, and 22 were observed with relatively fewer significant correlations (1–6 pairs; *p* < 0.05).

The above-mentioned results show that the volatile compounds in lemon juice vesicles at different drying stages were very different, and the samples could be classified in terms of their volatile compound profiles to specify these differences. The results provide the basic information for the process of drying lemon juice vesicles.

### 3.2. Changes in Nonvolatile Flavor Compounds during Drying Processes

#### 3.2.1. Nonvolatile Flavor Compounds and Contents in Lemon Juice Vesicles during Drying

Amino acids, organic acids, and soluble sugars are regarded as important nonvolatile components in fruits that affect their flavor, taste, and nutritional value [43,44]. The components and content of amino acids, organic acids, and soluble sugars in lemon juice vesicles during the drying processes of IFD, CFD, and AD are shown in Table 2.

Fourteen types of amino acids were detected in lemon juice vesicles during the drying processes. Specifically, 11 types were detected in the fresh sample; 13, 11, 10, and 9 types were detected in the samples that underwent IF for 0.5 h, IFD for 4 h, IFD for 8 h, and IFD for 12 h, respectively; 8, 8, 8, and 7 types were detected in samples that underwent CF for 4 h, CFD for 4 h, CFD for 8 h, and CFD for 12 h, respectively; and 7, 6, and 7 were detected for the samples of AD for 2 h, AD for 4 h, and AD for 6 h, respectively. The amino acid types in the samples generally decreased as the three drying methods progressed. The final dried samples after IFD for 12 h, CFD for 12 h, and AD for 6 h lost three, four, and five types of original amino acids, respectively, when compared with the fresh samples. Meanwhile, glycine, threonine, and isoleucine were the new amino acid components introduced in the samples during the drying processes. IFD contributed to a greater number of amino acid types in the samples than CFD and AD, which was beneficial to achieving a better flavor quality in the final dried product.

Concerning the concentration of amino acids, the main amino acids present at a high content were serine, alanine, tyrosine, and aspartic acid, in descending order. Complex changes were observed in the content profiles of amino acids in the samples during the drying processes, presenting different variation trends for both the different types of amino acids and the different drying methods. In terms of IFD, the total content of amino acids sharply decreased after IF for 0.5 h, then slightly increased during the subsequent FD stages, which mainly involved the variation in serine content. As for CFD, the total content of amino acids also sharply decreased after CF for 4 h, then slightly increased after CFD for 4 h, followed by a slight decrease after CFD for 8 h and CFD for 12 h. During AD, the total content of amino acids sharply decreased after AD for 2 h. This was followed by an obvious increase after AD for 4 h and AD for 6 h, which was mainly attributed to the variation in glutamate and serine. During IF for 0.5 h and CF for 4 h, the ultra-structure of the tissue cells could be damaged due to the growth of ice crystals, promoting the ensuing release of the relevant enzymes and pro-oxidants to enable protein denaturation and destruction. This could account for the reduction in amino acid content at the freezing stages. It had been confirmed by Wang et al. that the rapid freezing treatment of IF caused reduced damage to the tissue cells in samples when compared with that of the relatively slow freezing treatment of CF [45]. This could account for the fact that the notably greater reduction in amino acids occurred in the samples of CF for 4 h when compared with those of IF for 0.5 h. The decrease, increase, and disappearance of amino acids during the dehydration stages of the three methods could be caused by the degradation of the proteins or amino acids due to the complicated conditions of temperature, enzyme activity, and moisture content, which may be partially attributed to the Maillard and oxidation reactions involved with substances such as amino acids, sugars, and oxygen. Regarding the final dried products of the three methods, the highest total content of amino acids was in the samples of AD for 6 h, followed by the samples of IFD for 12 h and CFD for 12 h, which were mainly dominated by changes in the contents of glutamate and serine.

Three types of organic acids were detected during the drying processes: malic acid, ascorbic acid, and citric acid. Of these, citric acid was the most abundant one determining the variation trends in the total content of organic acids. A similar decreasing trend was observed both in the contents of ascorbic acid and citric acid during the drying processes of the three methods. In contrast, a complicated increasing/decreasing trend was observed in the content of malic acid within the samples during drying. In terms of the final dried products of the three methods, the total content of organic acids in the sample after CFD for 12 h was significantly higher than those of IFD for 12 h and AD for 6 h.

The main soluble sugars in the juice vesicles detected during the drying processes were fructose, glucose, sucrose, and maltose. The content of maltose showed a fluctuating trend with decreases and increases during IFD, CFD, and AD. Additionally, a similar decreasing trend was observed in the contents of all the other three soluble sugars. Regarding the final dried products of the three methods, the total content of soluble sugars in the samples of AD for 6 h was significantly higher than those of IFD for 12 h and CFD for 12 h. As for the total content of amino acids, organic acids, and soluble sugars in the samples of the final dried products, the highest one was in AD for 6 h, followed by those of CFD for 12 h and IFD for 12 h, with significant differences (*p* < 0.05).

#### 3.2.2. PCA, Cluster Analysis, and Correlation Analysis of the Nonvolatile Flavor Compounds

**Figure 3:**
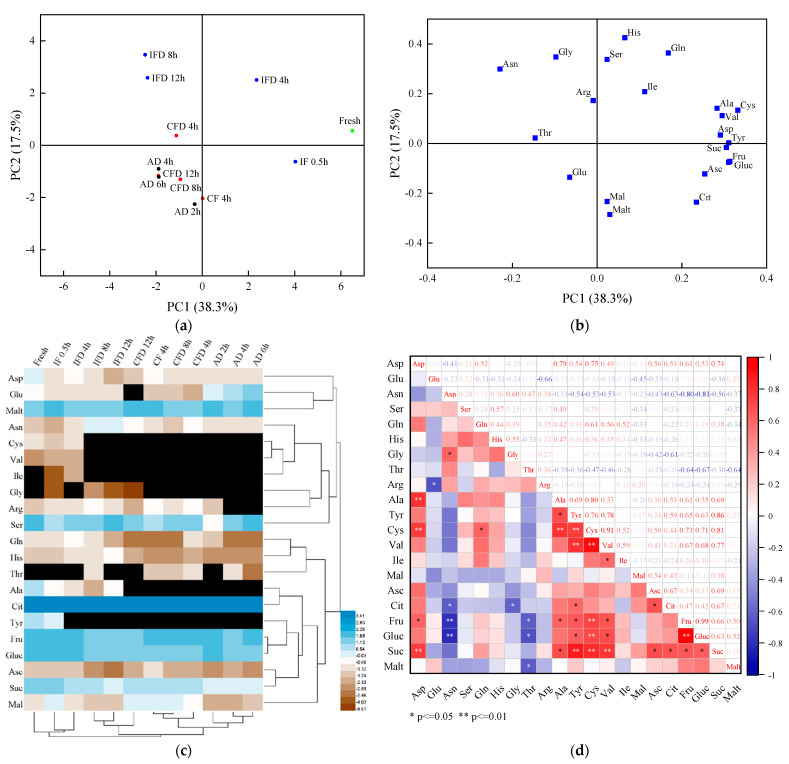
Principal component analysis, hierarchical cluster analysis, and Pearson’s correlation analysis based on the contents of nonvolatile flavor compounds in the samples during the drying processes. The abbreviated words represent the nonvolatile flavor compounds in Table 2. (**a**): Score plot of principal component analysis of the different drying stages; (**b**): Score plot of principal component analysis of the nonvolatile flavor compounds; (**c**): Heatmap of hierarchical cluster analysis; (**d**): Heatmap of Pearson’s correlation analysis.

The PCA results of the profiles of the nonvolatile compounds of the juice vesicles during the drying processes are shown in Figure 3a,b. The accumulative variance contribution rate of the first PC (38.3%) and the second PC (17.5%) was 55.8%. Figure 3a illustrates that the samples of fresh and IF for 0.5 h could be distinguished by higher positive values of PC1; the samples of IFD for 4 h, IFD for 8 h, and IFD for 12 h could be distinguished by higher positive values of PC2; and the samples of other drying stages could be distinguished by smaller negative values of PC1 and PC2. Figure 3b illustrated that the detected nonvolatile compounds could be divided into several groups. In particular, the higher positive values of PC1 could be defined by Ala, Cys, Val, Asp, Tyr, Suc, Fru, Gluc, Asc, and Cit.

The HCA results of the profiles of nonvolatile compounds of the juice vesicles during the drying processes are shown in Figure 3c. The samples at different drying stages could be classified into three main clusters. The first cluster included fresh, IF for 0.5 h, and IFD for 4 h samples. The second cluster included IFD for 8 h, IFD for 12 h, and CFD for 12 h samples. The samples at other drying stages were grouped into the third cluster. Nonvolatile compounds could be divided into three principal clusters. The first cluster included Asp, Glu, and Malt. The second cluster included Asn, Cys, Val, Ile, Gly, Arg, and Ser, while the other nonvolatile compounds were grouped into the third cluster.

The results of the correlation analysis for the 21 nonvolatile compounds are shown in Figure 3d. More than four significant positive correlations (*p* < 0.05) were observed in the pairs involving Asp, Ala, Tyr, Cys, Val, Fru, Gluc, and Suc. In addition, Asn and Thr both presented significant negative correlations (*p* < 0.05) with three nonvolatile compounds, which showed that the content changes among these nonvolatile compounds are contradictory. Ser, His, and Mal presented no significant correlations with other nonvolatile compounds.

#### 3.2.3. Correlation Analysis of the Volatile and Nonvolatile Flavor Compounds

**Figure 4:**
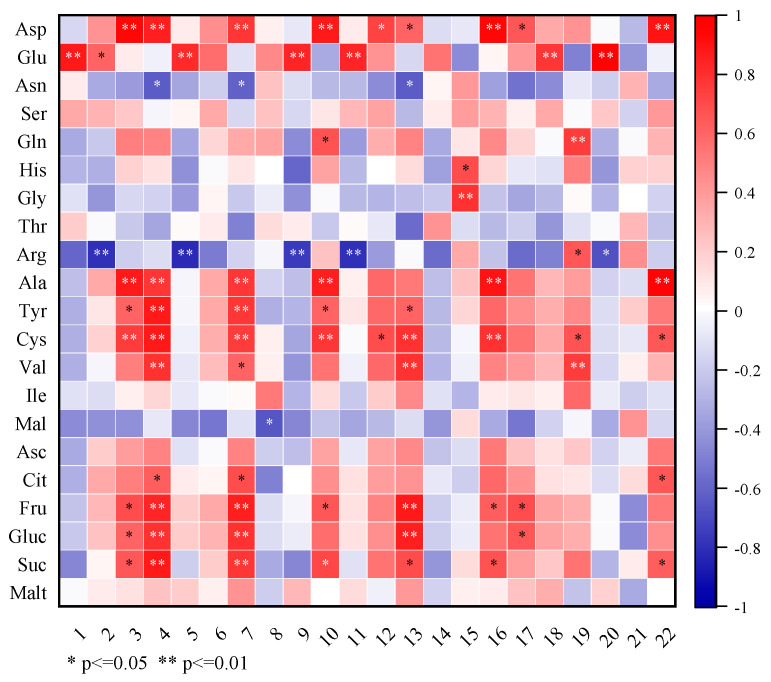
Pearson’s correlation analysis based on the contents of volatile and nonvolatile flavor compounds in the samples during the drying processes. Numbers 1~22 are the corresponding codes for the 22 kinds of volatile compounds in Table 1. The abbreviated words represent the nonvolatile flavor compounds in Table 2.

A correlation analysis was carried out to demonstrate the relation between the volatile and nonvolatile compounds (Figure 4). It was found that Arg and Asn presented significant negative correlations with five and three volatile compounds, respectively. Many nonvolatile compounds including Asp, Glu, Ala, Tyr, Cys, Val, Fru, Gluc, and Suc presented significant positive correlations with more than four of the volatile compounds. According to the results in Figure 4, the correlation analysis demonstrated that most of the amino acids and the soluble sugars were closely related to the profiles of volatile compounds in the lemon juice vesicle samples during the drying processes, whereas the organic acids were rarely correlated with the volatile compounds, except for citric acid (which had three significant correlation coefficients).

The changes in the volatile flavor compounds in food during drying are considered to represent an extremely complex physical and chemical process, which is influenced by many factors. It is well known that flavor precursors play a decisive role in the formation of food flavor compounds. Various amino acids, proteins, reducing sugars, and carbohydrates are regarded as important flavor precursors in processed foods [46]. We deduced that some reactions involved with the above-mentioned nonvolatile compounds occurred during the drying processes, thereby accounting for the chemical mechanism of the formation of the volatile profiles in our experiments.

It has been verified that a series of organic compounds and volatile compounds with low carbon numbers in various fruits and vegetables, including alcohols, aldehydes, acids, and esters, are mostly derived from the synthesis of amino acids through the involvement of related enzymes (such as deaminase, decarboxylase, dehydrogenase, and ester synthase) [47,48]. In addition, under the conditions of heating and drying, amino acids and sugars can undergo thermal degradation, the Maillard reaction, and lipid oxidation, thereby generating volatile flavor compounds of alcohols, aldehydes, and esters [49,50]. In summary, the combined complex effect of enzymatic, oxidative, and heating reactions involved with those flavor precursors may contribute to the final profiles of volatile and nonvolatile flavor compounds in lemon juice vesicles during the three drying processes of IFD, CFD, and AD.

## 4. Conclusions

This study investigated the changes in the volatile and nonvolatile flavor compounds in lemon juice vesicles during the processes of IFD, CFD, and AD. Compared with the fresh samples, the final dried products of IFD, CFD, and AD lost seven, seven, and six volatile flavor compounds, respectively; their total volatile compound contents were 0.80 ± 0.07, 0.48 ± 0.05, and 1.98 ± 0.12 mg/g, respectively. The content of volatile compounds of the lemon juice vesicles during IFD and CFD presented a descending trend, which was fast but that then became slow. However, those during AD presented an initially decreasing and then increasing trend. For the final dried samples, IFD, CFD, and AD resulted in the loss of three, four, and five amino acids, respectively; their total amino acid contents were 9.44 ± 0.25, 5.29 ± 0.10, and 10.83 ± 0.20 mg/g, respectively; their total organic acid contents were 37.93 ± 0.47, 45.94 ± 0.34, and 38.07 ± 0.39 mg/g, respectively; and their total soluble sugar contents were 15.88 ± 0.25, 15.63 ± 0.20, and 17.12 ± 0.20 mg/g, respectively. In addition, significant positive or negative correlations were observed among various flavor compounds, demonstrating that most of the amino acids and the soluble sugars were closely related to the profiles of volatile compounds in the samples of lemon juice vesicles during drying.

## Figures and Tables

**Table 1:** Volatile flavor compounds and content of lemon juice vesicles for different drying methods/(mg/g FW).

Compound Category	Code Number	Compound Name	Retention Time/Min	Retention Index ^(a)^	Retention Index ^(b)^	Match Score/%	Fresh Sample	IFD				CFD				AD		
								IF 0.5 h	IFD 4 h	IFD 8 h	IFD 12 h	CF 4 h	CFD 4 h	CFD 8 h	CFD 12 h	AD 2 h	AD 4 h	AD 6 h
Alkenes	1	β-Myrcene	12.24	932	947	95	0.02 ± 0.01 ^b^	0.01 ± 0.00 ^b^	0.03 ± 0.02 ^b^	0.04 ± 0.03 ^b^	0.03 ± 0.02 ^b^	0.01 ± 0.01 ^b^	0.03 ± 0.01 ^b^	0.01 ± 0.00 ^b^	0.02 ± 0.01 ^b^	0.01 ± 0.01 ^b^	0.10 ± 0.02 ^a^	0.13 ± 0.03 ^a^
	2	α-Terpinene	13.55	941	966	95	0.05 ± 0.00 ^a^	ND ^b^	0.01 ± 0.00 ^b^	ND ^b^	ND ^b^	ND ^b^	ND ^b^	ND ^b^	0.01 ± 0.00 ^b^	0.01 ± 0.00 ^b^	0.06 ± 0.00 ^a^	0.04 ± 0.03 ^a^
	3	D-limonene	14.17	993	986	92	0.52 ± 0.02 ^a^	0.23 ± 0.01 ^b^	0.22 ± 0.02 ^bc^	0.18 ± 0.01 ^bcd^	0.12 ± 0.02 ^d^	0.20 ± 0.03 ^bc^	0.16 ± 0.02 ^cd^	0.06 ± 0.01 ^e^	0.05 ± 0.01 ^e^	0.23 ± 0.03 ^b^	0.20 ± 0.05 ^bc^	0.17 ± 0.03 ^bcd^
	4	γ-Terpinene	15.04	1027	1014	96	0.27 ± 0.03 ^a^	0.22 ± 0.01 ^b^	0.12 ± 0.02 ^c^	0.06 ± 0.01 ^de^	0.02 ± 0.01 ^ef^	0.14 ± 0.02 ^c^	0.06 ± 0.02 ^de^	0.04 ± 0.01 ^def^	0.01 ± 0.00 ^f^	0.12 ± 0.01 ^c^	0.07 ± 0.02 ^d^	0.06 ± 0.01 ^de^
	5	Bisa bolene	36.38	1518	1504	91	0.02 ± 0.01 ^bc^	0.01 ± 0.00 ^c^	0.01 ± 0.00 ^c^	ND ^c^	ND ^c^	0.01 ± 0.00 ^c^	ND ^c^	ND ^c^	ND ^c^	0.02 ± 0.01 ^bc^	0.06 ± 0.03 ^a^	0.05 ± 0.03 ^ab^
Subtotal							0.88 ± 0.07 ^a^	0.47 ± 0.03 ^b^	0.39 ± 0.06 ^bc^	0.28 ± 0.04 ^cde^	0.17 ± 0.03 ^ef^	0.36 ± 0.04 ^bcd^	0.25 ± 0.03 ^de^	0.11 ± 0.02 ^f^	0.09 ± 0.03 ^f^	0.39 ± 0.04 ^bc^	0.49 ± 0.07 ^b^	0.45 ± 0.09 ^b^
Alcohols	6	Linalool	17.84	1082	1052	90	0.05 ± 0.01 ^ab^	0.04 ± 0.01 ^abc^	0.03 ± 0.00 ^bc^	0.04 ± 0.02 ^abc^	0.02 ± 0.01 ^bc^	0.02 ± 0.01 ^bc^	0.02 ± 0.01 ^bc^	0.01 ± 0.00 ^c^	0.01 ± 0.00 ^c^	0.02 ± 0.01 ^bc^	0.06 ± 0.00 ^a^	0.05 ± 0.02 ^ab^
	7	β-Terpineol	20.235	1158	1145	92	0.05 ± 0.01 ^a^	0.03 ± 0.00 ^b^	0.01 ± 0.00 ^c^	ND ^c^	ND ^c^	ND ^c^	ND ^c^	ND ^c^	ND ^c^	0.03 ± 0.01 ^b^	ND ^c^	ND ^c^
	8	Terpinen-4-ol	21.50	1137	1163	88	0.06 ± 0.02 ^cde^	0.05 ± 0.01 ^cde^	0.17 ± 0.06 ^a^	0.10 ± 0.04 ^abc^	0.07 ± 0.01 ^cde^	0.01 ± 0.00 ^de^	0.12 ± 0.01 ^abc^	ND ^e^	0.04 ± 0.02 ^cde^	0.08 ± 0.02 ^bcd^	0.11 ± 0.04 ^abc^	0.15 ± 0.04 ^ab^
	9	(-)-Terpinen-4-ol	21.73	1137	1175	89	0.06 ± 0.00 ^b^	0.02 ± 0.01 ^b^	0.03 ± 0.00 ^b^	0.03 ± 0.01 ^b^	0.03 ± 0.01 ^b^	0.06 ± 0.01 ^b^	0.07 ± 0.00 ^b^	0.02 ± 0.01 ^b^	0.06 ± 0.02 ^b^	0.13 ± 0.05 ^a^	0.15 ± 0.04 ^a^	0.17 ± 0.03 ^a^
	10	α-Terpineol	22.45	1143	1175	95	0.51 ± 0.10 ^a^	0.26 ± 0.05 ^bc^	0.26 ± 0.03 ^bc^	0.23 ± 0.03 ^bc^	0.18 ± 0.02 ^bcd^	0.21 ± 0.03 ^bc^	0.22 ± 0.03 ^bc^	0.15 ± 0.02 ^cde^	0.07 ± 0.01 ^e^	0.27 ± 0.04 ^bc^	0.10 ± 0.02 ^de^	0.07 ± 0.01 ^e^
	11	Citronellol	24.09	1179	1227	90	0.03 ± 0.00 ^b^	0.02 ± 0.00 ^bc^	ND ^c^	ND ^c^	0.01 ± 0.00 ^bc^	ND ^c^	ND ^c^	ND ^c^	ND ^c^	ND ^c^	0.07 ± 0.01 ^a^	0.07 ± 0.03 ^a^
	12	Nerol	25.20	1228	1210	91	0.22 ± 0.03 ^a^	0.16 ± 0.02 ^bc^	0.14 ± 0.01 ^bc^	0.07 ± 0.03 ^de^	0.04 ± 0.01 ^de^	0.14 ± 0.03 ^bc^	0.10 ± 0.03 ^cd^	0.04 ± 0.01 ^de^	0.03 ± 0.01 ^e^	0.04 ± 0.01 ^de^	0.15 ± 0.04 ^bc^	0.15 ± 0.03 ^bc^
Subtotal							0.98 ± 0.11 ^a^	0.58 ± 0.09 ^bc^	0.64 ± 0.08 ^bc^	0.47 ± 0.06 ^bcd^	0.35 ± 0.04 ^de^	0.44 ± 0.07 ^cd^	0.53 ± 0.09 ^bcd^	0.22 ± 0.04 ^e^	0.21 ± 0.03 ^e^	0.57 ± 0.08 ^bc^	0.64 ± 0.11 ^bc^	0.66 ± 0.10 ^bc^
Aldehydes	13	Octanal	12.96	1005	957	86	0.01 ± 0.00 ^a^	0.01 ± 0.01 ^a^	0.01 ± 0.00 ^a^	ND ^a^	ND ^a^	0.01 ± 0.00 ^a^	ND ^a^	ND ^a^	ND ^a^	0.01 ± 0.00 ^a^	ND ^a^	ND ^a^
	14	Nonanal	18.08	1104	1084	92	0.02 ± 0.00 ^c^	0.01 ± 0.00 ^c^	0.03 ± 0.01 ^c^	0.03 ± 0.00 ^c^	0.02 ± 0.01 ^c^	0.02 ± 0.01 ^c^	0.04 ± 0.01 ^c^	0.01 ± 0.00 ^c^	0.02 ± 0.01 ^c^	0.02 ± 0.01 ^c^	0.18 ± 0.04 ^a^	0.08 ± 0.02 ^b^
	15	Decanal	23.09	1204	1202	91	0.01 ± 0.00 ^a^	0.01 ± 0.00 ^a^	ND ^a^	0.02 ± 0.00 ^a^	0.02 ± 0.00 ^a^	ND ^a^	ND ^a^	0.01 ± 0.00 ^a^	0.01 ± 0.00 ^a^	ND ^a^	ND ^a^	ND ^a^
	16	Neral	24.56	1174	1238	88	0.18 ± 0.03 ^a^	0.06 ± 0.02 ^bc^	0.07 ± 0.02 ^b^	0.04 ± 0.01 ^bcd^	0.03 ± 0.01 ^cd^	0.05 ± 0.01 ^bc^	0.05 ± 0.01 ^bc^	0.01 ± 0.00 ^d^	0.03 ± 0.01 ^cd^	0.06 ± 0.03 ^bc^	0.05 ± 0.01 ^bc^	0.05 ± 0.01 ^bc^
	17	Citral	25.96	1174	1241	94	0.51 ± 0.08 ^a^	0.31 ± 0.04 ^b^	0.30 ± 0.03 ^b^	0.16 ± 0.02 ^c^	0.14 ± 0.01 ^cd^	0.34 ± 0.02 ^b^	0.14 ± 0.01 ^cd^	0.10 ± 0.01 ^cd^	0.08 ± 0.01 ^d^	0.34 ± 0.02 ^b^	0.46 ± 0.07 ^a^	0.31 ± 0.04 ^b^
	18	Perilla aldehyde	26.20	1207	1280	90	0.08 ± 0.01 ^b^	0.06 ± 0.02 ^bc^	0.04 ± 0.01 ^c^	ND ^d^	ND ^d^	0.02 ± 0.01 ^d^	ND ^d^	ND ^d^	ND ^d^	0.01 ± 0.00 ^d^	ND ^d^	0.17 ± 0.03 ^a^
Subtotal							0.81 ± 0.08 ^a^	0.46 ± 0.07 ^c^	0.45 ± 0.05 ^c^	0.25 ± 0.03 ^d^	0.21 ± 0.03 ^d^	0.44 ± 0.05 ^c^	0.23 ± 0.02 ^d^	0.13 ± 0.02 ^e^	0.14 ± 0.02 ^e^	0.44 ± 0.05 ^c^	0.69 ± 0.10 ^b^	0.61 ± 0.09 ^b^
Esters	19	Geranyl isovalerate	23.81	1586	1597	86	0.05 ± 0.01 ^bc^	0.07 ± 0.03 ^ab^	0.08 ± 0.02 ^a^	0.03 ± 0.02 ^bc^	0.04 ± 0.01 ^bc^	0.01 ± 0.00 ^d^	0.06 ± 0.01 ^ab^	0.02 ± 0.01 ^cd^	ND ^d^	0.03 ± 0.02 ^bc^	ND ^d^	ND ^d^
	20	Lavandulyl acetate	30.11	1272	1283	89	0.04 ± 0.02 ^c^	0.03 ± 0.01 ^c^	0.04 ± 0.01 ^c^	0.02 ± 0.01 ^c^	ND ^d^	0.04 ± 0.01 ^c^	0.03 ± 0.01 ^c^	ND ^d^	0.02 ± 0.01 ^c^	0.03 ± 0.02 ^c^	0.12 ± 0.03 ^b^	0.26 ± 0.05 ^a^
	21	Diiso butyl phthalate	49.27	1908	1871	92	ND ^e^	0.04 ± 0.01 ^ab^	ND ^e^	ND ^e^	0.03 ± 0.00 ^bc^	ND ^e^	0.06 ± 0.02 ^a^	0.01 ± 0.01 ^de^	0.02 ± 0.01 ^cd^	ND ^e^	ND ^e^	ND ^e^
Subtotal							0.09 ± 0.04 ^cd^	0.14 ± 0.03 ^b^	0.12 ± 0.02 ^bc^	0.05 ± 0.02 ^de^	0.07 ± 0.02 ^de^	0.05 ± 0.01 ^de^	0.15 ± 0.03 ^b^	0.03 ± 0.01 ^e^	0.04 ± 0.01 ^de^	0.06 ± 0.02 ^de^	0.12 ± 0.03 ^bc^	0.26 ± 0.05 ^a^
Ketones	22	Carvone	24.76	1234	1248	87	0.02 ± 0.00 ^a^	ND ^b^	ND ^b^	ND ^b^	ND ^b^	ND ^b^	ND ^b^	ND ^b^	ND ^b^	ND ^b^	ND ^b^	ND ^b^
Total							2.78 ± 0.16 ^a^	1.65 ± 0.13 ^cd^	1.60 ± 0.09 ^cd^	1.05 ± 0.04 ^f^	0.80 ± 0.07 ^g^	1.29 ± 0.11 ^e^	1.16 ± 0.09^ef^	0.49 ± 0.06^h^	0.48 ± 0.05 ^h^	1.46 ± 0.07 ^d^	1.94 ± 0.10 ^b^	1.98 ± 0.12 ^b^

Note: Retention index ^(a)^ is achieved from the experiments. Retention index ^(b)^ is reported in [1,4,19,20,21,22]. “ND” indicates that is not detected or below detection limit. “FW” means fresh weight. Data points in the same row with different letters are significantly different (*p* < 0.05).

**Table 2:** Components and content of amino acids, organic acids, and soluble sugars of lemon juice vesicles through different drying methods/(mg/g FW).

Compound Category	Compound Name	Fresh Sample	IFD				CFD				AD		
			IF 0.5 h	IFD 4 h	IFD 8 h	IFD 12 h	CF4 h	CFD 4 h	CFD 8 h	CFD 12 h	AD 2 h	AD 4 h	AD 6 h
Amino acids	Aspartic acid (Asp)	1.37 ± 0.05 ^a^	0.54 ± 0.04 ^c^	0.68 ± 0.10 ^b^	0.53 ± 0.04 ^c^	0.14 ± 0.05 ^e^	0.72 ± 0.03 ^b^	0.48 ± 0.05 ^cd^	0.38 ± 0.01 ^d^	0.23 ± 0.01 ^e^	0.44 ± 0.01 ^cd^	0.39 ± 0.04 ^d^	0.42 ± 0.06 ^cd^
	Glutamate (Glu)	0.70 ± 0.03 ^d^	0.32 ± 0.02 ^ef^	0.40 ± 0.09 ^e^	0.32 ± 0.00 ^ef^	0.38 ± 0.05 ^e^	0.18 ± 0.07 ^fg^	0.14 ± 0.03 ^g^	0.20 ± 0.02 ^fg^	ND ^h^	1.09 ± 0.04 ^c^	1.72 ± 0.06 ^b^	4.19 ± 0.13 ^a^
	Asparagine (Asn)	0.21 ± 0.02 ^gh^	0.13 ± 0.01 ^h^	0.42 ± 0.00 ^def^	1.09 ± 0.01 ^a^	0.98 ± 0.03 ^ab^	0.54 ± 0.06 ^d^	0.95 ± 0.01 ^b^	0.23 ± 0.08 ^gh^	0.68 ± 0.09 ^c^	0.32 ± 0.04 ^fg^	0.51 ± 0.02 ^de^	0.41 ± 0.09 ^ef^
	Serine (Ser)	6.36 ± 0.10 ^a^	2.70 ± 0.03 ^g^	5.09 ± 0.04 ^c^	5.06 ± 0.03 ^c^	6.16 ± 0.05 ^b^	2.58 ± 0.03 ^h^	3.53 ± 0.03 ^f^	3.60 ± 0.05 ^ef^	4.07 ± 0.04 ^d^	0.64 ± 0.02 ^i^	4.09 ± 0.04 ^d^	3.66 ± 0.06 ^e^
	Glutamine (Gln)	0.43 ± 0.01 ^b^	0.31 ± 0.00 ^c^	0.55 ± 0.06 ^a^	0.54 ± 0.06 ^a^	0.10 ± 0.09 ^de^	0.05 ± 0.00 ^e^	0.36 ± 0.01 ^bc^	0.05 ± 0.00 ^e^	0.04 ± 0.01 ^e^	0.18 ± 0.05 ^d^	0.04 ± 0.00 ^e^	0.05 ± 0.02 ^e^
	Histidine (His)	0.30 ± 0.04 ^b^	0.28 ± 0.04 ^b^	0.31 ± 0.04 ^b^	0.32 ± 0.02 ^b^	0.59 ± 0.03 ^a^	0.06 ± 0.00 ^d^	0.13 ± 0.00 ^c^	0.06 ± 0.00 ^d^	0.11 ± 0.00 ^cd^	0.06 ± 0.00 ^d^	0.06 ± 0.00 ^d^	0.06 ± 0.00 ^d^
	Glycine (Gly)	ND ^e^	0.02 ± 0.00 ^c^	ND ^e^	0.07 ± 0.01 ^a^	0.03 ± 0.00 ^b^	ND ^e^	ND ^e^	ND ^e^	0.01 ± 0.00 ^d^	ND ^e^	ND ^e^	ND ^e^
	Threonine (Thr)	ND ^e^	ND ^e^	ND ^e^	0.31 ± 0.02 ^b^	ND ^e^	0.23 ± 0.02 ^c^	0.47 ± 0.01 ^a^	0.21 ± 0.01 ^c^	ND ^e^	ND ^e^	ND ^e^	0.04 ± 0.00 ^d^
	Arginine (Arg)	0.20 ± 0.01 ^g^	0.35 ± 0.03 ^e^	0.53 ± 0.02 ^cd^	0.59 ± 0.06 ^bc^	0.48 ± 0.01 ^d^	0.76 ± 0.01 ^a^	0.65 ± 0.03 ^b^	0.51 ± 0.04 ^d^	0.15 ± 0.00 ^g^	0.27 ± 0.05 ^f^	ND ^h^	ND ^h^
	Alanine (Ala)	2.42 ± 0.14 ^a^	0.62 ± 0.05 ^b^	0.40 ± 0.06 ^c^	0.15 ± 0.05 ^d^	0.58 ± 0.08 ^b^	ND ^d^	ND ^d^	ND ^d^	ND ^d^	ND ^d^	ND ^d^	ND ^d^
	Tyrosine (Tyr)	1.85 ± 0.07 ^a^	1.51 ± 0.04 ^b^	ND ^c^	ND ^c^	ND ^c^	ND ^c^	ND ^c^	ND ^c^	ND ^c^	ND ^c^	ND ^c^	ND ^c^
	Cystine (Cys)	0.43 ± 0.01 ^a^	0.30 ± 0.00 ^c^	0.33 ± 0.00 ^b^	ND ^d^	ND ^d^	ND ^d^	ND ^d^	ND ^d^	ND ^d^	ND ^d^	ND ^d^	ND ^d^
	Valine (Val)	0.08 ± 0.01 ^c^	0.14 ± 0.01 ^a^	0.11 ± 0.01 ^b^	ND ^d^	ND ^d^	ND ^d^	ND ^d^	ND ^d^	ND ^d^	ND ^d^	ND ^d^	ND ^d^
	Isoleucine (Ile)	ND ^c^	0.02 ± 0.00 ^b^	0.16 ± 0.01 ^a^	ND ^c^	ND ^c^	ND ^c^	ND ^c^	ND ^c^	ND ^c^	ND ^c^	ND ^c^	ND ^c^
Subtotal		14.35 ± 0.23 ^a^	7.24 ± 0.15 ^e^	8.98 ± 0.17 ^d^	8.98 ± 0.19 ^d^	9.44 ± 0.25 ^c^	5.12 ± 0.10 ^h^	6.71 ± 0.09 ^g^	5.24 ± 0.06 ^h^	5.29 ± 0.10 ^h^	3.00 ± 0.07 ^i^	7.15 ± 0.12 ^f^	10.83 ± 0.20 ^b^
Organic acids	Malic acid (Mal)	0.33 ± 0.02 ^c^	1.11 ± 0.08 ^a^	0.31 ± 0.03 ^c^	0.24 ± 0.02 ^de^	0.20 ± 0.03 ^de^	1.18 ± 0.01 ^a^	0.68 ± 0.05 ^b^	1.31 ± 0.09 ^a^	1.29 ± 0.07 ^a^	0.14 ± 0.05 ^e^	0.15 ± 0.05 ^e^	0.28 ± 0.06 ^cd^
	Ascorbic acid (Asc)	0.28 ± 0.04 ^ab^	0.19 ± 0.06 ^bcd^	0.23 ± 0.08 ^abc^	0.06 ± 0.01 ^ef^	0.05 ± 0.01 ^ef^	0.31 ± 0.06 ^a^	0.11 ± 0.03 ^de^	0.14 ± 0.02 ^cde^	0.15 ± 0.03 ^cde^	0.09 ± 0.01 ^def^	0.12 ± 0.02 ^de^	0.09 ± 0.00 ^def^
	Citric acid (Cit)	53.37 ± 0.17 ^a^	46.93 ± 0.65 ^b^	37.10 ± 0.58 ^e^	37.42 ± 0.55 ^e^	37.68 ± 0.50 ^e^	46.71 ± 0.55 ^b^	44.22 ± 1.00 ^cd^	43.53 ± 0.80 ^cd^	44.50 ± 0.30 ^c^	42.55 ± 0.54 ^d^	42.79 ± 0.42 ^d^	37.70 ± 0.34 ^e^
Subtotal		53.98 ± 0.20 ^a^	48.23 ± 0.62 ^b^	37.64 ± 0.65 ^e^	37.72 ± 0.51 ^e^	37.93 ± 0.47 ^e^	48.20 ± 0.59 ^b^	47.01 ± 1.07 ^d^	44.98 ± 0.83 ^c^	45.94 ± 0.34 ^c^	42.78 ± 0.56 ^d^	43.06 ± 0.46 ^d^	38.07 ± 0.39 ^e^
Soluble sugars	Fructose (Fru)	8.36 ± 0.07 ^a^	7.62 ± 0.19 ^b^	6.81 ± 0.26 ^c^	4.67 ± 0.13 ^f^	4.95 ± 0.30 ^ef^	5.68 ± 0.22 ^d^	5.94 ± 0.08 ^d^	5.71 ± 0.14 ^d^	4.20 ± 0.06 ^g^	7.31 ± 0.21 ^b^	5.36 ± 0.15 ^de^	5.48 ± 0.17 ^d^
	Glucose (Gluc)	6.14 ± 0.18 ^a^	6.06 ± 0.23 ^a^	5.53 ± 0.19 ^b^	4.01 ± 0.06 ^ef^	4.30 ± 0.15 ^de^	4.69 ± 0.17 ^c^	4.74 ± 0.09 ^c^	4.78 ± 0.12 ^c^	3.81 ± 0.05 ^f^	5.69 ± 0.14 ^b^	4.51 ± 0.17 ^cd^	4.58 ± 0.12 ^cd^
	Sucrose (Suc)	3.85 ± 0.12 ^a^	3.78 ± 0.09 ^a^	2.36 ± 0.07 ^c^	1.82 ± 0.01 ^e^	1.31 ± 0.01 ^g^	2.72 ± 0.13 ^b^	2.70 ± 0.04 ^b^	2.08 ± 0.09 ^d^	1.99 ± 0.04 ^d^	1.56 ± 0.06 ^f^	1.39 ± 0.05 ^g^	1.34 ± 0.05 ^g^
	Maltose (Malt)	5.26 ± 0.01 ^c^	5.88 ± 0.22 ^b^	4.79 ± 0.12 ^de^	5.05 ± 0.09 ^cd^	5.32 ± 0.20 ^c^	5.95 ± 0.07 ^b^	4.99 ± 0.10 ^de^	4.69 ± 0.11 ^e^	5.63 ± 0.09 ^b^	6.33 ± 0.09 ^a^	4.91 ± 0.22 ^de^	5.72 ± 0.06 ^b^
Subtotal		23.61 ± 0.24 ^a^	23.34 ± 0.30 ^a^	19.49 ± 0.24 ^c^	15.55 ± 0.19 ^e^	15.88 ± 0.25 ^e^	19.04 ± 0.31 ^c^	18.37 ± 0.15 ^f^	17.26 ± 0.28 ^d^	15.63 ± 0.20 ^e^	20.89 ± 0.29 ^b^	16.17 ± 0.32 ^e^	17.12 ± 0.20 ^d^
Total		91.94 ± 0.72 ^a^	78.81 ± 0.69 ^b^	66.11 ± 0.74 ^d^	62.25 ± 0.67 ^e^	63.25 ± 0.73 ^e^	72.36 ± 0.80 ^c^	70.09 ± 0.96 ^c^	67.48 ± 0.91 ^d^	66.86 ± 0.61 ^d^	66.67 ± 0.64 ^d^	66.38 ± 0.85 ^d^	66.02 ± 0.68 ^d^

Note: “ND” indicates that is not detected or below detection limit. “FW” means fresh weight. Data points in the same row with different letters are significantly different (*p* < 0.05).

## Data Availability

Not applicable.
